# Cross-frequency coupling within and between the human thalamus and neocortex

**DOI:** 10.3389/fnhum.2013.00084

**Published:** 2013-03-25

**Authors:** Thomas H. B. FitzGerald, Antonio Valentin, Richard Selway, Mark P. Richardson

**Affiliations:** Department of Clinical Neurosciences, Institute of Psychiatry, King's College LondonLondon, UK

**Keywords:** thalamus, neocortex, cross-frequency coupling, oscillations, synchrony

## Abstract

There is currently growing interest in, and increasing evidence for, cross-frequency interactions between electrical field oscillations in the brains of various organisms. A number of theories have linked such interactions to crucial features of neuronal function and cognition. In mammals, these interactions have mostly been reported in the neocortex and hippocampus, and it remains unexplored whether similar patterns of activity occur in the thalamus, and between the thalamus and neocortex. Here we use data recorded from patients undergoing thalamic deep-brain stimulation for epilepsy to demonstrate the existence and prevalence, across a range of frequencies, of both phase–amplitude (PAC) and amplitude–amplitude coupling (AAC) both within the thalamus and prefrontal cortex (PFC), and between them. These cross-frequency interactions may play an important role in local processing within the thalamus and neocortex, as well as information transfer between them.

## Introduction

Phase–amplitude coupling (PAC, often also called “nested oscillations”) occurs when the amplitude of an oscillation at a particular frequency is modulated by the phase of a lower frequency oscillation. This kind of cross-frequency coupling has been the subject of considerable recent interest (Canolty and Knight, 2010), as providing a mechanism for, amongst other things, working memory (Jensen and Lisman, 1998), spatial exploration (Lisman and Buzsaki, 2008), and visual perception (VanRullen and Koch, 2003; Palva and Palva, 2007).

Considerable experimental evidence has now been provided for PAC, but this has come mostly from work in animals (Bragin et al., 1995; Steriade et al., 1996; Chrobak and Buzsaki, 1998; Csicsvari et al., 2003; Cunningham et al., 2003; Lakatos et al., 2005). Some studies have explored PAC in humans, but these have been restricted to non-invasive recordings (Vanhatalo et al., 2004; Demiralp et al., 2007; Cohen et al., 2008; de Lange et al., 2008; Monto et al., 2008; Osipova et al., 2008) or recordings made using electrodes implanted in the mesial temporal lobe (Mormann et al., 2005; Axmacher et al., 2010), the nucleus accumbens (Cohen et al., 2009), or placed onto the neocortex (Canolty et al., 2006; Maris et al., 2011).

Amplitude–amplitude coupling (AAC) is another possible mechanism for cross-frequency interaction which has been considerably less well explored. Evidence has been found for it between the theta (4–8 Hz) and gamma (31–70 Hz) bands during a spatial memory task in rats (Shirvalkar et al., 2010), and between delta (1–3 Hz) and gamma (31–70 Hz) bands in different regions of the occipital cortex in data recorded from subdural electrodes in humans (Bruns and Eckhorn, 2004).

Few studies have directly tested either cross-frequency coupling within the thalamus, or cross-frequency interactions between the thalamus and neocortex, which may play an important role in coordinating activity between brain regions. Staudigl et al. report evidence of PAC between beta oscillations in the thalamus and gamma oscillations in the neocortex during performance of a recognition memory task (Staudigl et al., 2012), whilst a recent paper also found evidence of cross-frequency coupling in the pulvinar nucleus of the macaque (Saalmann et al., 2012). We thus sought to test for PAC and AAC locally within the thalamus and neocortex, between low frequencies in the thalamus and high frequencies in the cortex (thalamo-cortical coupling), and between low frequencies in the cortex and high frequencies in the thalamus (cortico-thalamic coupling).

To address these questions, we used data recorded from 3 patients with intractable epilepsy who had thalamic deep brain stimulators targeted at the centromedian nucleus (Velasco et al., 2001), and subdural electrodes simultaneously implanted over the prefrontal cortex (PFC). We tested for the existence of PAC and AAC within both structures, as well as between them. We hypothesized that both regions would show strong evidence of PAC and AAC across a broad range of frequencies, and that significant coupling would also occur between the cortex and the thalamus.

## Materials and methods

### Subjects

**Figure 1:**
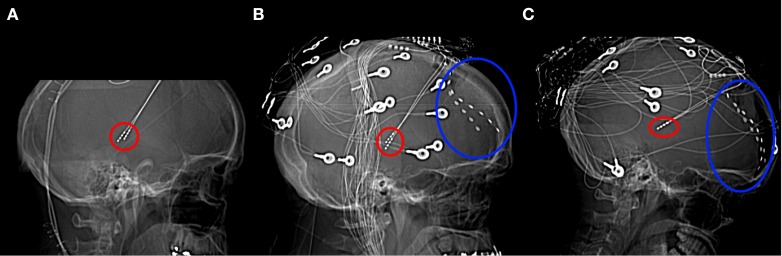
**Lateral x-radiographs from Patients 1 (A), 2 (B), and 3 (C).** Thalamic stimulator contacts are circled in red, subdural electrodes circled in blue. (Imaging for Patient 1 was carried out only after the removal of the subdural electrodes).

Data were recorded from 3 patients [Patient 1: male, 40 years (medication: clobazam, carbamazepine, levetiracetam, lamotrigine); Patient 2: male, 51 years (topiramate, levetiracetam, clobazam, pregabalin, carbamazepine); Patient 3: female, 18 years (lamotrigine, levetiracetam, clonazepam)] with intractable epilepsy who were undergoing experimental deep-brain stimulation therapy at King's College Hospital (UK). The experimental procedure was approved by the ethics committee of King's College Hospital. Bilateral four contact deep-brain stimulators (Medtronic Neurological Division, Minnesota, USA) were implanted, stereotactically targeted at the centromedian nucleus of the thalamus (Velasco et al., 2001) (Figure 1). In addition, two eight contact subdural strips were inserted onto the PFC to allow confirmation of thalamic electrode location according to the method of Velasco, which involves detection of a 6 Hz frontal recruiting rhythm in response to 6 Hz thalamic stimulation. The location of stimulators and subdural electrodes were confirmed by electrical stimulation and visualization of post-implantation CT. (Note that no claim is made here about which thalamic regions the electrical activity we report originates from).

### Recording and preprocessing

In 2 patients, recordings were made using a 96-channel Digital Lynx system (Neuralynx, Bozeman, Montana, USA) at a sampling rate of 32 kHz, filtered between 0.1 Hz and 9 kHz, while they performed a cued attention paradigm (Patients 1, 2), a memory task (Patient 2), and a Go-NoGo task (Patient 1) (total 10 recording sessions). In the remaining patient, recordings were taken during two sessions of quiet wakefulness with a sampling rate of 256 Hz on a NicoletOne recording system (Viasys Healthcare, San Diego, California, USA).

All data were recorded referenced either to a midline scalp electrode fitted where clinical dressings allowed, or, where such an electrode could not be fitted or was contaminated with excessive line noise, from one of the subdural electrodes. The data from Patients 1 and 2 were first resampled to 1024 Hz. The data were then low-pass filtered at 410 Hz (Sampling Rate/2.5) (Patients 1, 2) or 70 Hz (Patient 3) using a two-way least squares finite impulse response (FIR) filter implemented in the EEGlab (Delorme and Makeig, 2004) function *eegfilt*.

Six minute data segments were extracted from the beginning of each recording. These were visually inspected for artifacts or epileptiform activity, and any epochs containing these were discarded from further analysis. Noise-contaminated channels and those displaying persistent epileptiform activity were identified visually and by inspection of their power spectra, and these were also excluded from further analysis. To minimize the effects of line noise on the data the recordings were then notch-filtered at 50 Hz and the first two harmonics using *eegfilt*. To minimize volume conduction effects, the data were converted with a bipolar montage, with the time courses from adjacent electrodes subtracted from one another.

Patients 1 and 3 suffered no seizures during or within 24 h prior to the recording period. The relevant records for Patient 2 have not been kept, preventing us from making any statement about the temporal relationship between these recordings and seizure activity; however we note that Patient 2 was able to adequately perform a memory task during recording.

### Phase–amplitude coupling

To balance a comprehensive search against sensitivity, given the need for multiple comparison correction, we set our frequencies of interest as being low frequencies at 2 Hz intervals between 2 and 30 Hz, and high frequencies at 2 Hz intervals between 6 and 64 Hz. To minimize contamination between nearby frequency bands we only considered coupling between oscillations where the high frequency was greater than twice that of the low frequency, giving us a total of 240 frequency band pairs (FBPs). Data were filtered using *eegfilt* into 2 Hz bands, centered on the frequency of interest.

We tested for phase–amplitude comodulation using the normalized modulation index proposed by (Canolty et al., 2006). Here a composite complex-valued signal *C* is created for all time points *t*, by combining *P*_*X*_ (*f*_1_, *t*), the instantaneous phase of the signal *X* at frequency *f*_1_, with *A*_*Y*_(*f*_2_, *t*), the amplitude envelope of signal *Y* at a higher frequency *f*_2_. Thus, at any time point *t*:

(1) $$C(f_{1},f_{2},t)=A_{Y}(f_{2},t)\exp\left(iP_{X}(f_{1},t)\right)$$

Because any radial asymmetry in *C* over and above that generated by the non-uniform distribution of *P*_*X*_(*f*_1_, *t*) can be attributed to mutual information between *P*_*X*_(*f*_1_, *t*) and *A*_*Y*_(*f*_2_, *t*), a convenient measure of coupling between the phase and amplitude signals can be derived from *C* simply by comparing the absolute value of its mean (the length of the resultant vector) to a surrogate distribution generated by offsetting *P*_*X*_(*f*_1_, *t*) and *A*_*Y*_(*f*_2_, *t*) by some large time lag (here taken to be a lag greater than at least half the period of an oscillation at the frequency of the lower edge of the pass-band); in other words, by normalizing it. The normalized modulation index is then the Z score of the mean of *C* assessed against a surrogate distribution generated by fitting a Gaussian to a distribution generated from 200 surrogate data sets, and *P*-values can be easily derived from this. PAC was regarded as significant when the relevant *P*-value was less than 0.05, false-discovery rate corrected for multiple comparisons across FBPs (Benjamini and Hochberg, 1995). The preferred phase (the phase at which the amplitude of the higher frequency oscillation is maximal) can be calculated by taking the angle of the resultant vector.

As in previous studies (Canolty et al., 2006; Kramer et al., 2008; Penny et al., 2008), these instantaneous phase and amplitude time series were created from an analytic signal derived by applying the Hilbert transform to data band-pass filtered within the frequency range of interest with an FIR filter implemented in EEGlab. Data points equivalent to the length of the filter order were removed from the beginning and end of the filtered time series to eliminate edge effects prior to further processing.

We tested for four kinds of PAC: local thalamic PAC, between oscillations recorded in the thalamus, cortical PAC between oscillations recorded in the cortex, thalamo-cortical PAC between low frequency (phase) oscillations recorded in the thalamus and high frequency (amplitude) oscillations recorded in the PFC, and cortico-thalamic PAC between low frequencies in the PFC and high frequencies in the thalamus.

Recently concerns have been raised that studies reporting nested oscillations might have done so because of the presence of sharp-edge artifacts in the data, rather than genuine PAC (Kramer et al., 2008). To minimize the risk of this we carried out a number of precautionary measures as recommended in that article. In addition to inspecting the raw data and excluding any epochs apparently containing sharp edges we plotted averaged data time-locked to the peaks of the high frequency amplitude envelope, and inspected frequency spectra and bicoherence plots for any signs of sharp-edge induced harmonics. No channels were found in which evidence of such artifacts could be discerned.

### Amplitude–amplitude coupling

To assess AAC, a normalized amplitude envelope correlation (NAE) was calculated (Bruns et al., 2000). Data were filtered and Hilbert transformed as described above. The correlation coefficient between the amplitude envelopes at different frequencies was then calculated. AAC significance was assessed against a surrogate distribution generated by shuffling the data and fitting a normal distribution as described above. Surrogate *P*-values were corrected for a two-tailed test. (This allowed us to consider cases where amplitude envelopes showed either strong positive or strong negative correlations.) NAEs were then normalized by subtracting the mean of the surrogate distribution of correlation coefficients, and dividing by the standard deviation.

### Additional analyses

#### PAC/phase

To determine whether, across the dataset, PAC tended to occur with some preferred phase, we calculated the preferred phase for each channel/FBP combination which showed significant PAC. We then calculated the circular average of these for each channel in each subject, pooled the resulting preferred phase estimates within each subject, and examined whether the data showed a pronounced peak using a Rayleigh test, which quantifies whether a circular data departs significantly from uniformity, assuming a Von Mises distribution (Fisher, 1996; Berens, 2009).

#### Comparing coupling types and locations

Correlation coefficients (*R*) were calculated between the proportion of channels showing PAC and AAC at each FBP for all four types of coupling (thalamic, cortical, thalamo-cortical, cortico-thalamic). We also tested the correlations between the proportion of thalamic and cortical channels showing PAC at each FBP, the proportion showing AAC, and the proportion of thalamo-cortical and cortico-thalamic channel pairs showing both types of coupling at each FBP. Significance was assessed using a permutation test in which one set of data was shuffled 1000 times and the correlation coefficient recalculated. Surrogate *P*-values were taken as the proportion of the surrogate *R* distribution which were greater than the observed value.

To compare the strength of coupling between thalamus and cortex, and between thalamo-cortical and cortico-thalamic interactions, we calculated the difference between the mean NMI and mean absolute NAE averaged over all sessions within a subject, channels or channel-pairs and FBPs, and assessed significance with a permutation test as described above.

#### Within-frequency amplitude to amplitude coupling

Additionally, we explored which frequency bands showed the strongest within frequency band amplitude to amplitude coupling between the thalamus and PFC. For each channel pair and frequency band from 2 to 64 Hz, the NAE was calculated and tested for significance as described above.

## Results

### Local thalamic cross-frequency coupling

**Figure 2:**
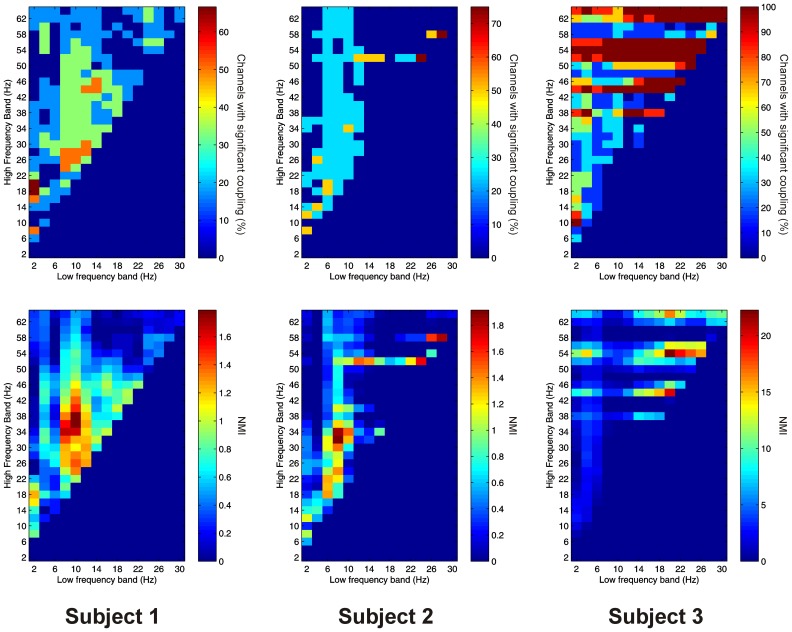
**Top row:** Proportion of channels showing significant local PAC in the thalamus (Subject 1 left, Subject 2 middle, Subject 3 right). **Bottom row:** Mean strength of PAC (assessed with the normalized modulation index) in the thalamus (Subject 1 left, Subject 2 middle, Subject 3 right).

Significant PAC was observed in the thalamus in all 3 subjects. Overall, 15 out of 16 channels (93.8%) showed significant PAC between at least one FBP. In both Patients 1 and 2, PAC was most common between low-frequency signals in the theta/alpha ranges and high-frequency signals in the beta/low gamma ranges (Figure 2), whilst in Patient 3 it was most common in beta/gamma FBPs (Figure 2).

**Figure 3:**
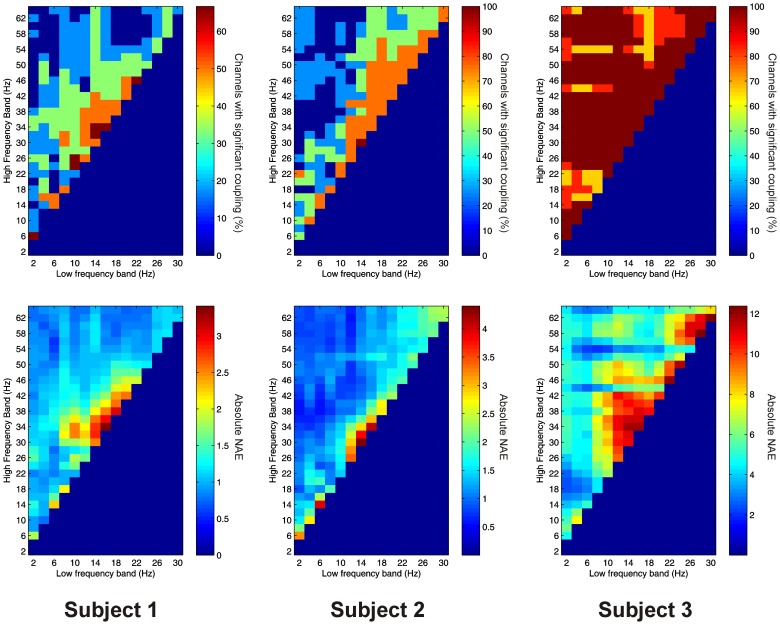
**Top row:** Proportion of channels showing significant local AAC in the thalamus (Subject 1 left, Subject 2 middle, Subject 3 right). **Bottom row:** Mean strength of AAC (assessed by the absolute value of the normalized amplitude envelope correlation) in the thalamus (Subject 1 left, Subject 2 middle, Subject 3 right).

Significant AAC was also observed in the thalamus in all 3 subjects, with 15 out of 16 channels (93.8%) showing significant AAC between at least one FBP. In all 3 subjects, AAC was most common between low frequencies in the alpha/beta ranges, and high frequencies in the low end of the gamma range (Figure 3), but significant AAC was also common between more widely spaced frequencies.

Subject 1 showed a strong positive correlation between the proportion of channels showing significant PAC and AAC at each FBP (*R* = 0.316, *P* < 0.001). Subjects 2 and 3 showed a strong negative correlation (Subject 2: *R* = −0.175, *P* = 0.010. Subject 3: *R* = −0.149, *P* = 0.016). Averaging across all subjects there was no significant correlation (*R* = −0.090, *P* = 0.170).

### Local prefrontal cross-frequency coupling

**Figure 4:**
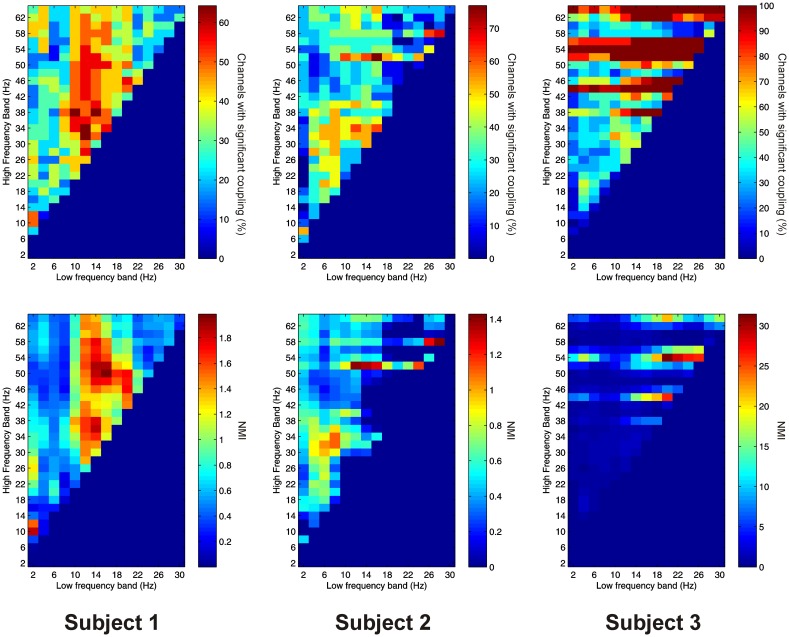
**Top row:** Proportion of channels showing significant local PAC in the PFC (Subject 1 left, Subject 2 middle, Subject 3 right). **Bottom row:** Mean strength of PAC (assessed with the normalized modulation index) in the PFC (Subject 1 left, Subject 2 middle, Subject 3 right).

Significant PAC was observed in the PFC in all 3 subjects. Overall, 41 out of 41 channels (100%) showed significant PAC between at least one FBP. In both Patients 1 and 2, PAC was most common between low-frequency signals in the theta/alpha ranges and high-frequency signals in the beta/gamma ranges (Figure 4), whilst in Patient 3 it was most common in beta/gamma FBPs (Figure 4).

**Figure 5:**
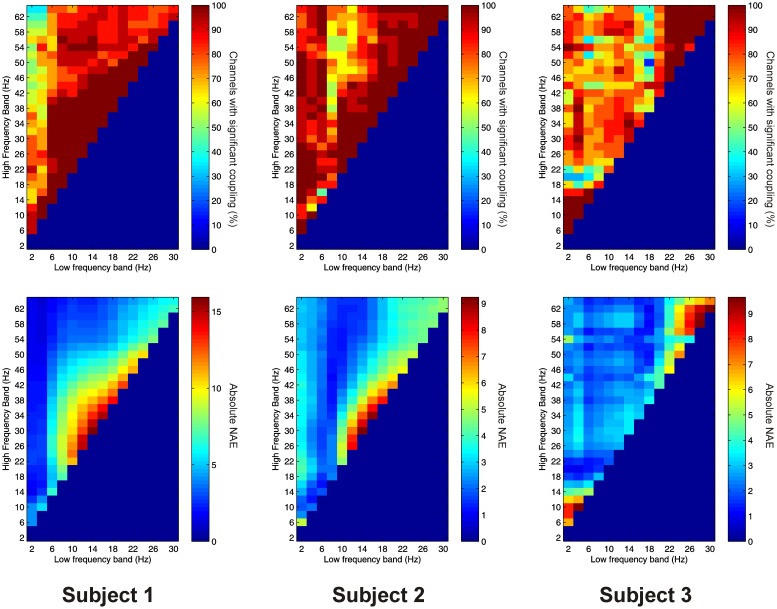
**Top row:** Proportion of channels showing significant local AAC in the PFC (Subject 1 left, Subject 2 middle, Subject 3 right). **Bottom row**: Mean strength of AAC (assessed by the absolute value of the normalized amplitude envelope correlation) in the PFC (Subject 1 left, Subject 2 middle, Subject 3 right).

Significant AAC was observed in the PFC in all 3 subjects, with 41 out of 41 channels (100%) showing significant AAC between at least one FBP. In all 3 subjects, AAC was common between low frequencies in the alpha/beta ranges, and high frequencies in the beta and gamma ranges (Figure 5). AAC was also common between low delta/theta ranges and high beta and gamma ranges in Subjects 2 and 3 (Figure 5).

Similar to the thalamus, Subject 1 showed a significant positive correlation between the proportion of channels showing significant PAC and AAC at each FBP (*R* = 0.435, *P* < 0.001), Subject 2 showed a negative correlation (*R* = −0.379, *P* < 0.001), and Subject 3 and the group average data showed no significant correlation (Subject 3: *R* = −0.106, *P* = 0.104. Group: *R* = 0.049, *P* = 0.454).

### Comparing local PAC and AAC profiles between thalamus and PFC

For PAC there was a significant positive correlation between the proportion of channels showing significant coupling at each FBP in the thalamus and cortex in all 3 subjects (Subject 1: *R* = 0.322, *P* < 0.001. Subject 2: *R* = 0.359, *P* < 0.001, Subject 3: *R* = 0.816, *P* < 0.001). AAC also showed a significant positive correlation in all subjects (Subject 1: *R* = 0.497, *P* < 0.001. Subject 2: *R* = 0.443, *P* < 0.001, Subject 3: *R* = 0.333, *P* < 0.001).

Both PAC and AAC were stronger in the cortex than the thalamus in Subject 1 (PAC: *T* − *C* = −0.321, *P* < 0.001. AAC: *T* − *C* = −4.284, *P* < 0.001). AAC was stronger in the cortex than the thalamus in Subject 2, and a trend toward stronger PAC in the cortex was also observed (PAC: *T* − *C* = −0.084, *P* = 0.080. AAC: *T* − *C* = −1.808, *P* < 0.001). AAC was significantly stronger in the thalamus than cortex in Subject 3 (*T* − *C* = 3.163, *P* < 0.001), but PAC was not significantly different (*T* − *C* = −0.653, *P* = 0.186).

### Coupling between low-frequency thalamic oscillations and high-frequency cortical oscillations

**Figure 6:**
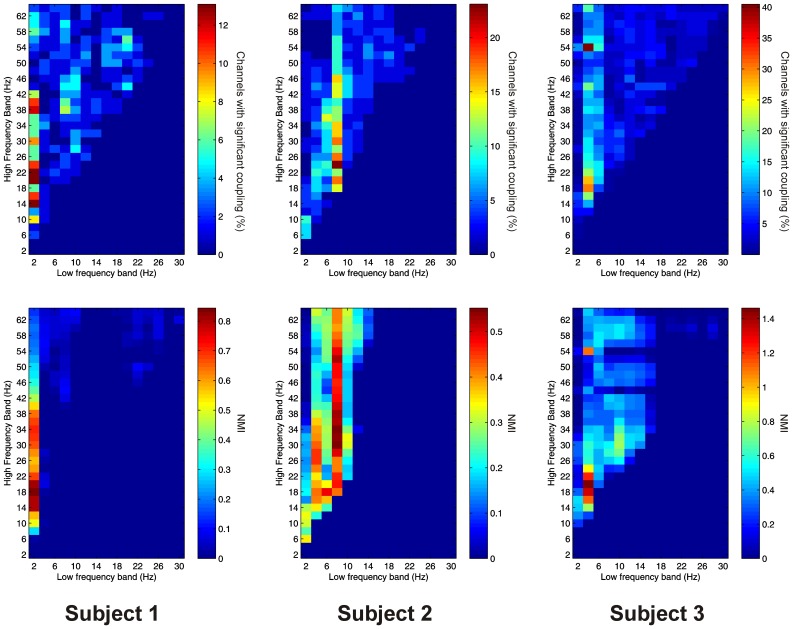
**Top row:** Proportion of channels showing significant thalamo-cortical PAC (coupling between a low-frequency in the thalamus and a high-frequency in the cortex) (Subject 1 left, Subject 2 middle, Subject 3 right). **Bottom row:** Mean strength of thalamo-cortical PAC (assessed with the normalized modulation index) (Subject 1 left, Subject 2 middle, Subject 3 right).

Significant PAC between low frequencies in the thalamus and high frequencies in the PFC (“thalamo-cortical” PAC) was observed in 144 out of 220 (65.5%) channel pair combinations, and was present in all subjects considered individually. PAC was most prevalent in all subjects between low-frequencies in the delta to alpha ranges and high frequencies in the beta to gamma ranges (Figure 6).

**Figure 7:**
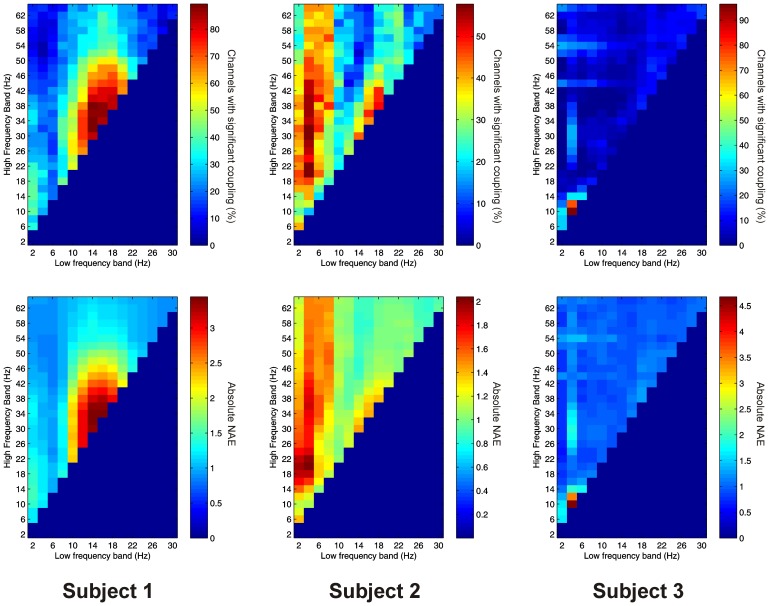
**Top row:** Proportion of channels showing significant thalamo-cortical AAC (coupling between a low-frequency in the thalamus and a high-frequency in the cortex) (Subject 1 left, Subject 2 middle, Subject 3 right). **Bottom row:** Mean strength of thalamo-cortical AAC (assessed by the absolute value of the normalized amplitude envelope correlation) (Subject 1 left, Subject 2 middle, Subject 3 right).

Thalamo-cortical AAC was observed in 217 out of 220 (98.6%) of channel pair combinations, and was present in all subjects. The FBPs showing the strongest thalamo-cortical AAC varied strongly between subjects (Figure 7).

Subject 2 showed a strong positive correlation between the proportion of channels showing significant PAC and AAC at each FBP (*R* = 0.205, *P* = 0.002). Subjects 1 and 2 showed no evidence of strong correlations (Subject 1: *R* = −0.002, *P* = 0.989; Subject 2: *R* = 0.096, *P* = 0.178).

### Coupling between low-frequency cortical oscillations and high-frequency thalamic oscillations

**Figure 8:**
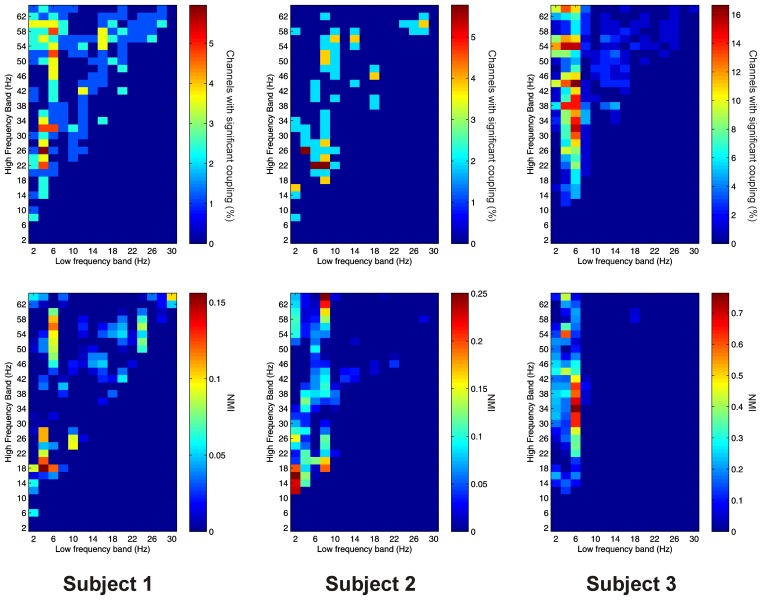
**Top row:** Proportion of channels showing significant cortico-thalamic PAC (coupling between a low-frequency in the PFC and a high-frequency in the thalamus) (Subject 1 left, Subject 2 middle, Subject 3 right). **Bottom row:** Mean strength of cortico-thalamic PAC (assessed with the normalized modulation index) (Subject 1 left, Subject 2 middle, Subject 3 right).

PAC between low frequencies in the PFC and high frequencies in the thalamus (“cortico-thalamic” PAC) was noticeably less prevalent than the other types we measured. Overall only 105 out of 220 (47.7%) channel pair combinations showed evidence of significant coupling. PAC was most prevalent in all subjects between low-frequencies in the delta to theta ranges and high frequencies in the beta to gamma ranges (Figure 8).

**Figure 9:**
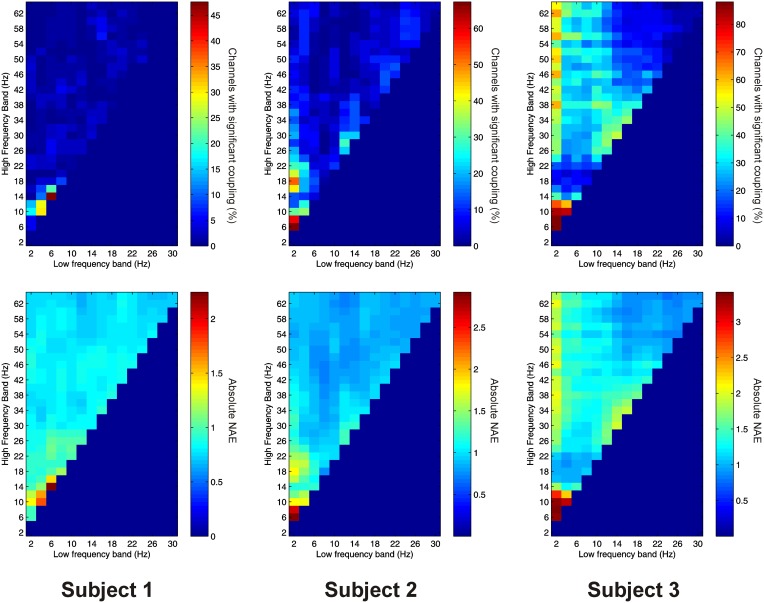
**Top row:** Proportion of channels showing significant cortico-thalamic AAC (coupling between a low-frequency in the PFC and a high-frequency in the thalamus) (Subject 1 left, Subject 2 middle, Subject 3 right). **Bottom row:** Mean strength of cortico-thalamic AAC (assessed by the absolute value of the normalized amplitude envelope correlation) (Subject 1 left, Subject 2 middle, Subject 3 right).

Cortico-thalamic AAC was observed in 198 out of 220 (90.0%) of channel pair combinations, and was present in all subjects. In all subjects it was concentrated at lower, and nearby frequencies, but AAC in other FBPs was also observed (Figure 9).

Subject 3 showed a strong positive correlation between the proportion of channels showing significant PAC and AAC at each FBP (*R* = 0.173, *P* = 0.020). Subjects 1 and 2 showed no evidence of strong correlations (Subject 1: *R* = −0.101, *P* = 0.096; Subject 2: *R* = −0.063, *P* = 0.316).

### Comparing distant thalamo-cortical and cortico-thalamic AAC and PAC

For PAC there was a significant positive correlation between the proportion of channel-pairs showing significant thalamo-cortical and cortico-thalamic coupling at each FBP in Subjects 2 and 3 (Subject 2: *R* = 0.370, *P* < 0.001; Subject 3: *R* = 0.635, *P* < 0.001). Subject 1 showed no evidence for a correlation (*R* = 0.033, *P* = 0.630). AAC showed a similar pattern across subjects. (Subject 1: *R* = −0.006, *P* = 0.906; Subject 2: *R* = 0.235, *P* < 0.001; Subject 3: *R* = 0.270, *P* = 0.002).

Comparing the strength of thalamo-cortical and cortico-thalamic coupling, we found that in both Subjects 2 and 3, thalamo-cortical PAC was significantly greater than cortico-thalamic (Subject 2: *TC* − *CT* = 0.082, *P* < 0.001. Subject 3: *TC* − *CT* = 0.279, *P* < 0.001). Subject 1 showed no significant difference (*TC* − *CT* = −0.025, *P* = 0.160). For AAC, Subjects 1 and 2 both showed significantly stronger thalamo-cortical coupling (Subject 1: *TC* − *CT* = 0.578, *P* < 0.001. Subject 2: *TC* − *CT* = 0.284, *P* < 0.001), whilst Subject 1 showed the opposite pattern (*TC* − *CT* = 0.255, *P* < 0.001).

### Preferred phase distributions

**Figure 10:**
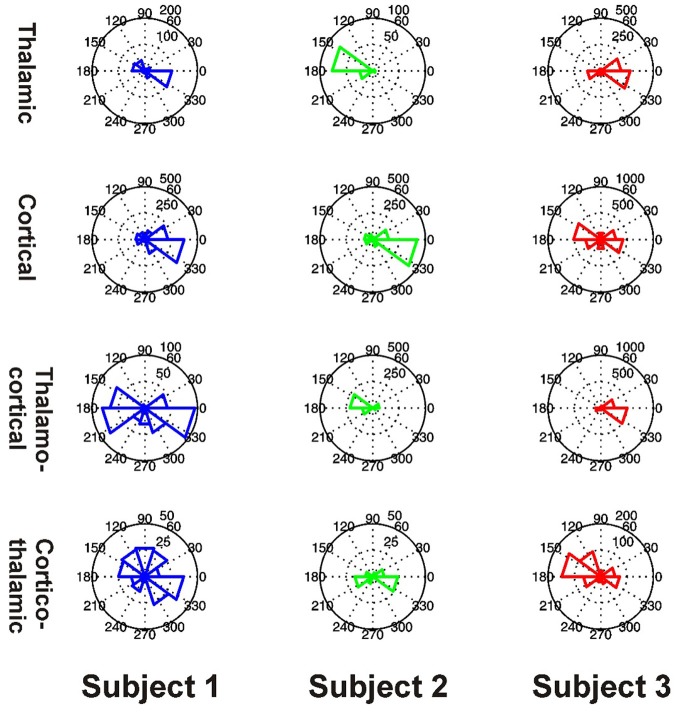
**Distributions of preferred PAC phases for FBPs which showed significant coupling in Subjects 1 (blue, left), 2 (green, middle), and 3 (red, right).** PAC types are shown separately as thalamic (top row), cortical (second row), thalamo-cortical (third row), and cortico-thalamic (fourth row) coupling.

All 3 subjects showed clear evidence of preferred phases for local PAC in the thalamus and PFC (all *P* < 0.001) (Figure 10). All 3 subjects showed clear evidence of preferred phases for thalamo-cortical PAC (all *P* < 0.001), but only Subjects 1 and 3 showed evidence of a preferred phase for cortico-thalamic PAC (Subject 1: *P* < 0.001, Subject 2: *P* = 0.160, Subject 3: *P* < 0.001).

### Amplitude–amplitude within frequency coupling

**Figure 11:**
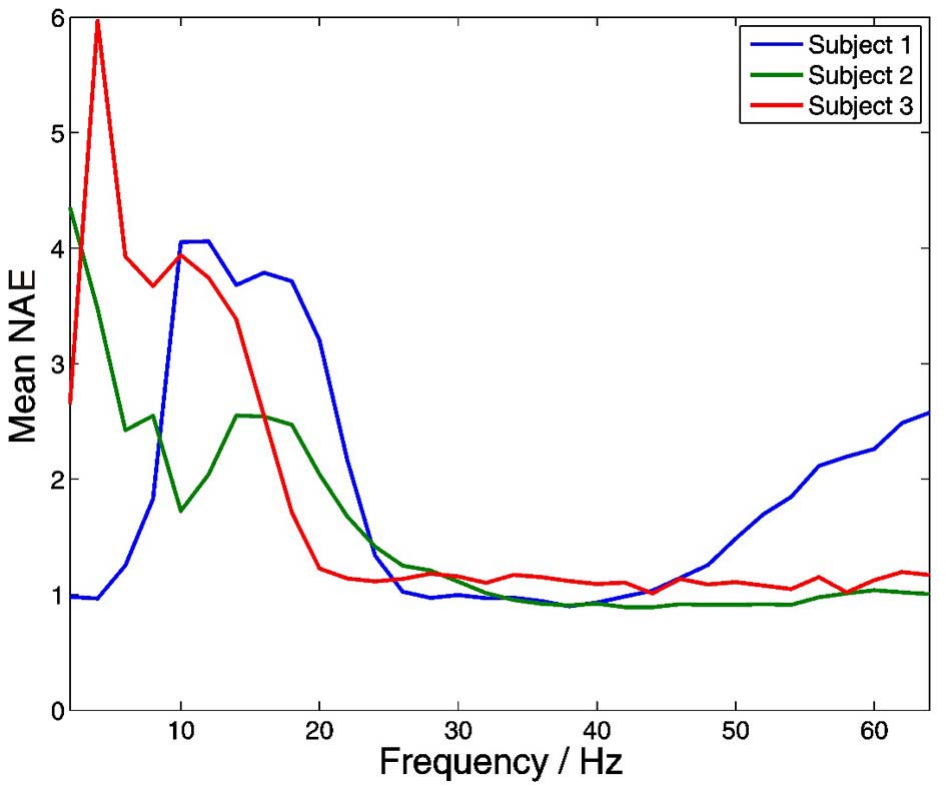
**Distributions of within-frequency amplitude co-modulation between the thalamus and PFC.** Mean coupling was positive in all subjects at all frequencies, and strongest at lower frequencies.

Significant within-frequency coupling between thalamus and PFC was found in 220 out of 220 (100%) of channel pairs. Across all frequencies and subjects, mean coupling was positive, and strongest at lower frequencies (Figure 11).

## Discussion

Overall, our data suggest that both PAC and AAC are prevalent features of neuronal activity across a range of frequencies within the thalamus and the neocortex, and that they also occur between them. Cross-frequency coupling is likely to be important for mediating interactions between neuronal activity at different spatial and temporal scales (Jensen and Colgin, 2007). Our findings add to the growing evidence for PAC and AAC in the brain (e.g., Canolty et al., 2006; Cohen et al., 2009; Axmacher et al., 2010; Shirvalkar et al., 2010; Maris et al., 2011), and suggest that neuronal activity in the thalamus, like that in the neocortex (Canolty et al., 2006; Maris et al., 2011), hippocampus (Axmacher et al., 2010), and striatum (Cohen et al., 2009) shows characteristic patterns of cross-frequency interaction. The fact that we also observe significant cross-frequency coupling between the thalamus and neocortex suggests that it may have an important role in controlling information flow between the thalamus and the neocortex (Sherman and Guillery, 2002; Timofeev and Bazhenov, 2005; Steriade, 2006), and perhaps between different cortical regions (Saalmann et al., 2012).

In general, we observed significant inhomogeneity between the subjects we measured in terms of the spectral profile and other properties of PAC and AAC. This may reflect a number of things, from individual differences in neuronal function, to slightly differing electrode locations, to the different cognitive demands placed upon the subject within the recording session. Unfortunately because of the small sample size, and the fact that we analyzed data recorded in several different situations, which differed across subjects, it is difficult to pin down the source of the variability. Our principal aim in presenting these data is to highlight the fact that these phenomena occur, and seem to be both widespread and variable in their occurrence, suggesting a flexible and potentially important role in local neuronal processing and coordination between regions.

Despite the high degree of between-subject variability, some patterns seem clear. In particular, our data suggest that there is a strong correlation between the strength of cross-frequency coupling expressed in an FBP in the thalamus, and that in the neocortex. This may reflect the fact that cross-frequency interactions reflect fundamental or state-dependent features of neuronal circuitry which are conserved across these structures, or alternatively it reflect the fact that coupling within and cross frequencies between the thalamus and neocortex is an important part of their interaction with one another. This interpretation is supported by the fact that thalamo-cortical and cortico-thalamic PAC and AAC were positively correlated across FBPs in both Subjects 2 and 3.

Within-frequency amplitude to amplitude coupling between the thalamus and neocortex was strongly present in our data, and was strongest at lower frequencies in all subjects. This fits with the notion that oscillations at lower frequency have a larger spatial extent, and thus play an important role in coordinating non-local activity (von Stein and Sarnthein, 2000).

A logical extension to this work would be to examine whether and how the various types of cross-frequency activity we observed are involved in cognition (Axmacher et al., 2010; Saalmann et al., 2012; Staudigl et al., 2012), and if so, when and how. It would also be interesting to see whether other kinds of local and non-local cross-frequency coupling occur (e.g., Palva et al., 2005; Darvas et al., 2009) within and between the thalamus and neocortex, potentially coordinating activity in these regions.

It must be borne in mind when considering these findings that they were obtained from the brains of epilepsy patients and thus may not precisely reflect neuronal activity in normal brains. We think this unlikely, given the catastrophic effects of disrupted thalamic activity in other contexts (Steinke et al., 1992), but it remains a possibility. One way to bolster the evidence for PAC and AAC in the thalamus would be to perform a similar analysis on data recorded from the thalami of healthy animals.

In this study we demonstrate that both phase–amplitude and AAC occur in the human thalamus, and between oscillations in the thalamus and neocortex. This supports the idea that cross-frequency coupling is a widespread and important feature of neuronal activity, and sheds light on possible mechanisms of information processing in the thalamus and thalamocortical communication.

### Conflict of interest statement

The authors declare that the research was conducted in the absence of any commercial or financial relationships that could be construed as a potential conflict of interest.
